# Dentin Sealing of Calcium Silicate-Based Sealers in Root Canal Retreatment: A Confocal Laser Microscopy Study

**DOI:** 10.3390/jfb13030114

**Published:** 2022-08-04

**Authors:** Blanca Ortiz-Blanco, José Luis Sanz, Carmen Llena, Adrián Lozano, Leopoldo Forner

**Affiliations:** Department of Stomatology, Faculty of Medicine and Dentistry, Universitat de València, 46010 Valencia, Spain

**Keywords:** bioceramic sealers, calcium silicate, retreatment, confocal laser microscopy

## Abstract

The aim of the present in vitro study was to evaluate the intratubular penetration of three bioceramic sealers in root canal retreatment. Here, 30 single-rooted human teeth were instrumented with the Protaper Universal system and filled with gutta-percha and the epoxy-resin-based sealer AH Plus mixed with rhodamine B. After two weeks in a humid environment, they were re-instrumented with Reciproc Blue and divided into three groups according to the endodontic sealer to be used in the re-filling (n = 10): G1: CeraSeal, G2: TotalFill BC Sealer, G3: TotalFill BC Sealer HiFlow. For the filling, a single cone technique was used, and the respective sealers were mixed with fluorescein. The roots were then sectioned at 2, 5, and 8 mm (apical, medial, and coronal measurement points, respectively) from the apex, and the dentinal tubule penetration depth and percentage of penetration around the canal perimeter were evaluated by means of confocal laser scanning microscopy (CLSM). Penetration between groups was compared using the Kruskal−Wallis test, and within each group using the Wilcoxon test. Statistical significance was established at *p* < 0.05. A non-significant reduction was found in the penetration depths and in a percentage of penetration around the canal perimeter between AH Plus and the tested calcium-silicate-based sealers (*p* > 0.05). Consequently, this reduction may not affect the three-dimensional seal of the root canal system in a negative manner. The penetration depth and percentage of penetration around the canal perimeter at both the root canal treatment and retreatment were significantly reduced from the coronal to apical points in all groups (*p* < 0.05).

## 1. Introduction

The field of endodontics is constantly evolving as a result of the introduction of new techniques and advances in the development of dental materials. Numerous types of sealers have been used together with gutta-percha for root canal filling, resulting in the development of new sealers with enhanced properties. In this regard, calcium-silicate-based or bioceramic materials were introduced as a novel category of sealers [1]. Bioceramic sealers possess a series of desirable biological properties, such as a high biocompatibility and a high pH [2,3]. From the perspective of material science, these sealers can be classified according to their main components into calcium-silicate-based sealers and calcium phosphate-based sealers [4]. Alternatively, epoxy-resin-based sealers have been the most widely used because of their low solubility, apical sealing ability, and adequate handling properties [5].

Currently, calcium-silicate-based sealers such as MTA Plus (Prevest Denpro GmbH, Heidelberg, Germany), MTA Fillapex (Angelus, Londrina, Brazil), iRoot SP (InnovativeBioceramix Inc., Vancouver, BC, Canada), and EndoSequence BC sealer (Brasseler USA, Savannah, GA, USA), also known as TotalFill BC Sealer (FKG, Munich, Germany) in Europe, have exhibited a low cytotoxicity, adequate bond strength, and adequate sealability. Additionally, TotalFill BC Sealer has been shown to enhance the osteoblastic differentiation of periodontal ligament cells, induce dentin remineralization, and possess antibacterial properties [6,7]. However, there are some concerns regarding TotalFill BC Sealer, as it may be affected by temperature, thus decreasing its flowability and setting time when heat is applied, which may negatively alter the quality of the filling when a heated filling technique is used, i.e., the continuous wave technique [8].

Recently, a new calcium-silicate-based sealer has been developed for this purpose, TotalFill BC Sealer HiFlow (FKG, Munich, Germany), whose physicochemical properties remain stable at temperatures that correspond to those achieved inside the root canal during root canal treatment with heated gutta-percha techniques. It also presents a lower viscosity and film thickness, and a higher flowability when heated [9]. Both TotalFill BC Sealer and TotalFill BC Sealer Hiflow have also shown adequate biological properties in laboratory studies and animal models [10,11].

On the other hand, CeraSeal (Meta Biomed Co., Cheongju, Korea) is a recently introduced pre-mixed endodontic sealer containing calcium silicates, zirconium oxide, and thickening agents. However, a limited number of studies have been published to date on this sealer, and there is a lack of evidence among the literature on its in vitro and in vivo properties [12].

One of the expected properties from these materials is their sealability, in other words, their potential to achieve a three-dimensional seal of the root canal system. As a means of assessing seal ability in vitro, the penetration depth inside dentinal tubules and the percentage of penetration around the canal perimeter can be evaluated. Confocal laser scanning microscopy (CLSM) is a widely used technique for the assessment of these parameters. In fact, several published studies use this method to evaluate the penetration of endodontic sealers [6,13,14].

However, to the best of the authors’ knowledge, there are no studies on the evaluation of the tubular penetration of bioceramic sealers in root canal retreatment. Root canal retreatment involves the removal of previous root canal fillings via chemical−mechanical disinfection through several methods, such as the use of solvents, Hedstroem files, ultrasonic tips, and/or rotary files. This procedure is considered as the treatment of choice in cases of inadequate initial root canal treatment, in the presence of signs and symptoms of periapical periodontitis (i.e., tenderness to percussion, localized swelling … etc.), in the presence of recurrent carious lesions, and/or in the presence inadequate (i.e., subject to microfiltration) provisional or definitive coronal restorations [15].

During this process, the three-dimensional cleaning of the endodontic space is the most critical phase, in order to reduce the bacterial load [16]. The presence of persistent polymicrobial microbiota, predominated by bacterial populations such as *E. faecalis* and *P. gingivalis,* may influence the clinical outcome of root canal treatment. Thus, root canal retreatment should focus on the disinfection and three-dimensional seal of the root canal system [17].

Achieving an adequate three-dimensional seal of the root canal system may be challenging in root canal retreatment, as remnants of the previous root canal sealer and or gutta-percha can remain both at a macroscopic and microscopic level, hindering the penetration of the new filling materials. The persistence of residual filling materials and the lack of penetration of the new filling materials may influence on the outcome of the root canal retreatment, due to the presence of persistent microbial populations and reinfection due to inadequate sealing [18].

Accordingly, the aim of the present in vitro study was to evaluate the tubular penetration of three bioceramic or calcium-silicate-based sealers in root canal retreatments in vitro. The null hypothesis was that there would be no differences between the tubular penetration of AH Plus in root canal treatment and the tubular penetration of the three calcium-silicate-based sealers in the root canal retreatment.

## 2. Materials and Methods

### 2.1. Sample Preparation

The protocol for this study was approved by the ethics committee from Universitat de València (ref. 85F5S1EEZ8322123). Thirty human maxillary incisors with mature apices extracted as a result of periodontal causes from healthy patients were selected for the present in vitro study, according to the following inclusion criteria: absence of carious lesions, root resorptions, pulpal calcifications, or other morphological anomalies. Prior informed consent was obtained from the donor patients. Two radiographic projections were performed for each tooth to confirm the presence of a single straight root canal. The teeth were stored in 0.1% thymol solution (37 °C, 100% humid environment) until processing and were de-coronated using a tungsten carbide disc (Komet Dental, Lemgo, Germany) at a low speed under refrigeration in order to obtain 16 mm long samples from the apex, which were stored in saline solution during the study.

### 2.2. Root Canal Instrumentation

Patency was confirmed in all specimens by means of K8 and K10 files (Denstply-Maillefer, Ballaigues, Switzerland) through visual inspection of the file’s tip at the apical foramen after insertion. The working length was established at 16−1 mm. All root canals were prepared using the Protaper Universal system up to F3 file (Dentsply-Maillefer, Ballaigues, Switzerland) with an X-Smart motor (Dentsply-Maillefer, Ballaigues, Switzerland) following the manufacturers’ instructions regarding speed and torque. The shaping sequence was performed following the manufacturer’s instructions (reaching working length and confirming glide path with manual files until K20 file, and then reaching working length with rotary files in the following order: X1, X2, F1, F2, and F3).

During the instrumentation process, patency was confirmed between each file, and 2.5% NaOCl was used as an irrigant solution. Once the preparation was completed, the roots of the experimental groups were irrigated with a final sequence of 5 mL of 17% EDTA for 3 min and 2 mL of 2.5% NaOCl, separated by saline solution. Root canals were then dried using F3 absorbent paper tips (Dentsply-Maillefer, Ballaigues, Switzerland).

### 2.3. Root Canal Filling

Root canal filling was performed by a previously trained single operator, via a single cone technique using F3 gutta-percha and epoxy-resin-based sealer AH Plus (Dentsply-Maillefer, Ballaigues, Switzerland). Gutta-percha cones were introduced until working length, 0.5 mm, and confirmed with periapical radiography. AH Plus was mixed manually and transported into the root canal using the gutta-percha cones. Then, 0.1% Rhodamine B (red) dye was added to the AH Plus sealer to provide the fluorescence that would allow the posterior microscopic evaluation. The samples were stored for 2 weeks at 100% humidity and 37 °C.

### 2.4. Root Canal Re-Treatment Instrumentation

The specimens were subjected to root canal retreatment using Reciproc Blue system (VDW, Munich, Germany). The files were used with VDW Silver motor (VDW, Munich, Germany), following the manufacturers’ instructions regarding speed and torque, and were introduced into the root canals in three forward and backward movements with light apical pressure until working length was reached. The root canals were then re-prepared using F4 and F5 instruments from the Protaper Universal system, as described above. Irrigation procedures were kept the same as in the initial endodontic treatment.

### 2.5. Root Canal Re-Treatment Filling

For root canal re-treatment filling, root samples (n = 30) were randomly allocated into the different experimental groups (n = 10 per group): CeraSeal (Meta Biomed Co., Cheongju, Korea), TotalFill BC Sealer (FKG, Munich, Germany), or TotalFill BC Sealer HiFlow (FKG, Munich, Germany). The composition of the tested sealers is presented in Table 1. A yellow dye (fluorescein) at a rate of 0.1% was incorporated into the different sealers to distinguish them from the dye used in the initial treatment (red). Root canal filling was performed by the same operator via a single cone technique using F5 gutta-percha and the respective stained sealers. Gutta-percha cones were introduced until the working length, 0.5 mm, and were confirmed with a periapical radiography. The pre-mixed sealers’ tips were introduced up to the medial−coronal third of the root canal and the sealer was injected into the root canal until visible at the root canal orifice. The samples were stored for 2 weeks at 100% humidity and 37 °C.

### 2.6. Confocal Laser Scanning Microscopy Assay (CLSM)

**Figure 1:**
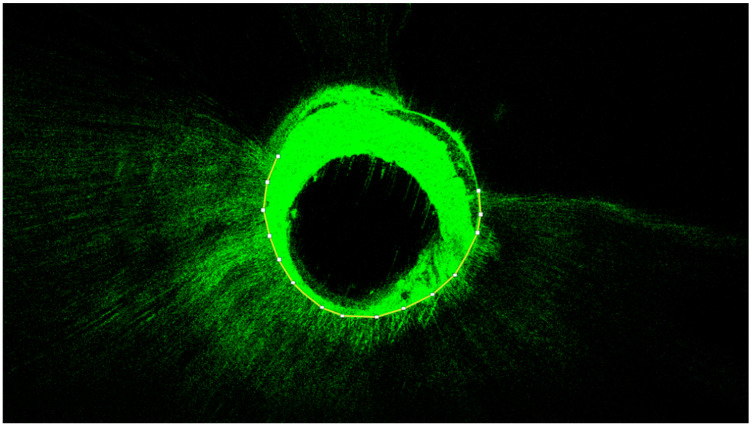
Representative CLSM image of the measurement of the sealer’s penetration around the canal perimeter. The central unstained portion corresponds to the gutta-percha. The surrounding, green-stained portion corresponds to the fluorescein-stained sealer. The prolongations of the green-stained portion correspond to the sealer penetration inside the dentinal tubules. The proportion of canal perimeter that showed signs of tubular penetration was marked and measured via ImageJ software (National Institutes of Health, Bethesda, MD, USA).

**Figure 2:**
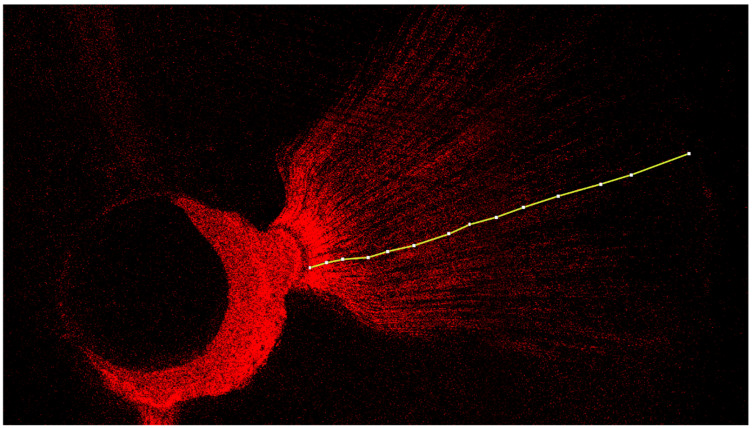
Representative CLSM image of the measurement of the sealer’s depth of penetration inside the dentinal tubules. The central unstained portion corresponds to the gutta-percha. The surrounding, red-stained portion corresponds to the rhodamine B-stained sealer. The prolongations of the red-stained portion correspond to the sealer penetration inside the dentinal tubules. The penetration depths were marked and measured via ImageJ software.

A diamond cutting disc (Komet Dental, Lemgo, Germany) was used at low speed to obtain three 1 mm thick discs of each root at 2, 5, and 8 mm from the apex of the tooth (apical, medial, and coronal measurement points, respectively). The equipment used for the microscopic study was a CLSM Leica TCS SP8 (Leica Microsystems GmbH, Wetzlar, Germany) from the Microscopy Service at Universitat de València (UCIM, Factultad de Medicina I Odontologia), with excitation and emission wavelengths for rhodamine B of 543 nm and 578 nm, and 514 nm for fluorescein staining. Sample analysis was performed at 10× magnification. The images were subsequently recorded and analyzed using ImageJ v1.53a software (National Institutes of Health, Bethesda, MD, USA). The tubular penetration depth (in μm) and the percentage of penetration around the canal perimeter were measured from the images obtained by CLSM, based on the methodology of a similar study [19,20] (Figure 1 and Figure 2).

### 2.7. Statistical Analysis

The obtained data were analyzed statistically using IBM^®^ SPSS V25.00 software (IBM^®^ SPSS Statistics, Inc., Chicago, IL, USA). The measurements of the tubular penetration depth and percentage of penetration around the canal perimeter were performed once by a single investigator for each of the samples. The descriptive data of tubular penetration depth and percentage of penetration around the canal perimeter of the residual sealer of the first root canal filling (AH Plus sealer) and of the sealer used in the retreatment (CeraSeal/TotalFill BC Sealer/TotalFill BC Sealer HiFlow) are presented. As the data did not possess a normal distribution, the mean and median values and the interquartile range (IQR) of the data are given. Both the percentages of penetration around the canal perimeter and the depth of penetration in micrometers for each sealer were compared between the different measurement points using the Kruskal−Wallis test. The Mann−Whitney U test was used to compare the groups globally and for each measurement point.

## 3. Results

### 3.1. Results for CeraSeal

**Figure 3:**
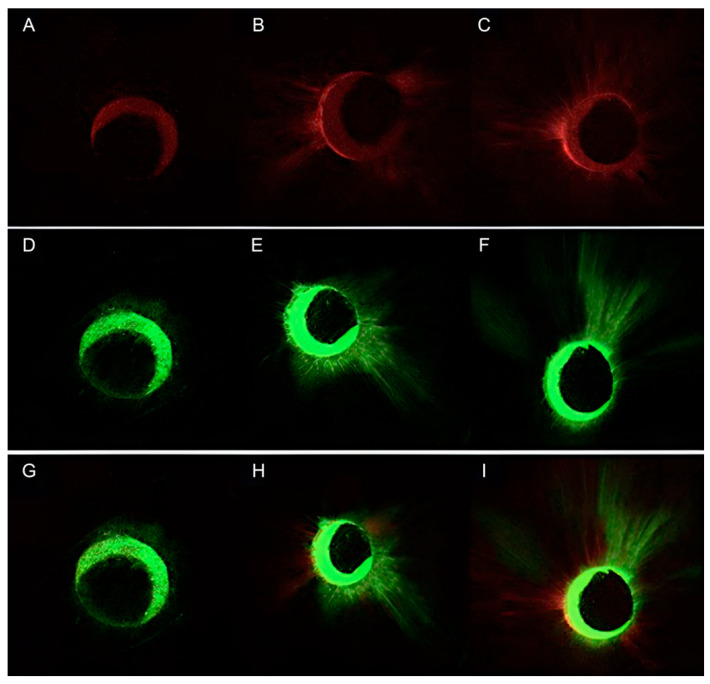
Representative CLSM images of the samples treated with gutta-percha and AH Plus and re-treated with gutta-percha and CeraSeal. AH Plus: apical point (**A**), medial point (**B**), coronal point (**C**); CeraSeal: apical point (**D**), medial point (**E**), coronal point (**F**); both sealers: apical point (**G**), medial point (**H**), coronal point (**I**).

Quantitative results of the CLSM image analysis in terms of the tubular penetration depth and the percentage of penetration around the canal perimeter exhibited by AH Plus and CeraSeal are presented in Table 2. The representative CLSM images of the results for AH Plus and CeraSeal are presented in Figure 3.

No significant differences were observed between the tubular penetration depth and percentage of penetration around the canal perimeter between AH Plus sealer and CeraSeal (*p* > 0.05), both in total and per third (apical, middle, or coronal).

**Figure 4:**
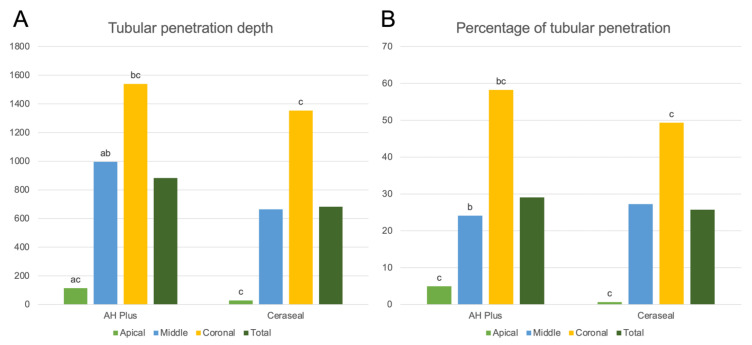
Graphical representation of the tubular penetration depth (**A**) and percentage of tubular penetration around the canal perimeter (**B**) for AH Plus and CeraSeal. (a) Significant difference between apical and middle measurement points within each sealer group (*p* < 0.05). (b) Significant difference between the middle and coronal measurement points within each sealer group (*p* < 0.05). (c) Significant difference between apical and coronal measurement points within each sealer group (*p* < 0.05).

AH Plus samples showed a significantly different depth of penetration in all measurement points, reducing from the coronal to the apical points (*p* < 0.05). The CeraSeal samples, however, only showed a significant reduction in the depth of penetration from the coronal to the apical measurement points (*p* < 0.05) (Figure 4A).

Regarding the percentage of tubular penetration, the AH Plus samples exhibited a significant difference between the coronal and apical points (*p* < 0.05) and the coronal and middle points (*p* < 0.05), while the CeraSeal samples only showed a significant reduction in the percentage of tubular penetration from the coronal to the apical measurement points (*p* < 0.05) (Figure 4B).

### 3.2. Results for TotalFill BC Sealer

**Figure 5:**
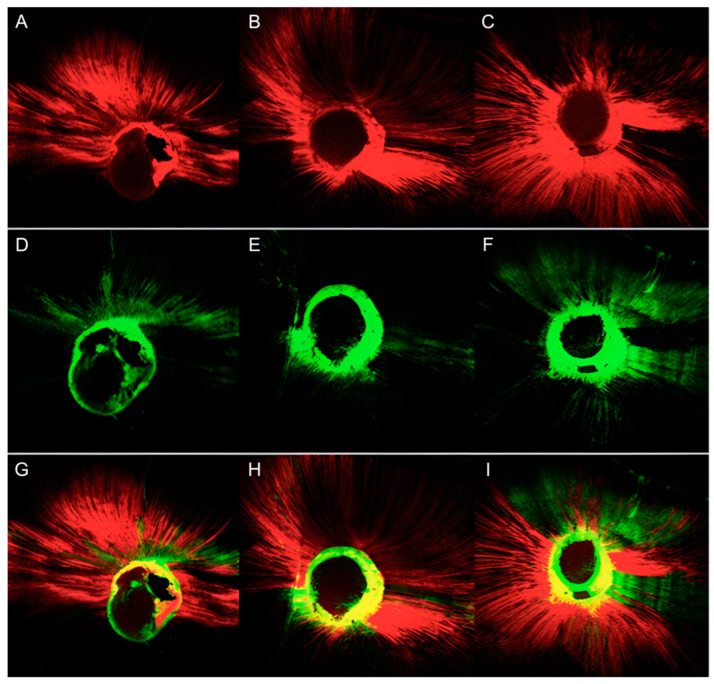
Representative CLSM images of the samples treated with gutta-percha and AH Plus and re-treated with gutta-percha and TotalFill BC Sealer. AH Plus: apical point (**A**), medial point (**B**), coronal point (**C**); TotalFill BC Sealer: apical point (**D**), medial point (**E**), coronal point (**F**); both sealers: apical point (**G**), medial point (**H**), coronal point (**I**).

The quantitative results of the CLSM image analysis in terms of the tubular penetration depth and percentage of penetration around the canal perimeter exhibited by AH Plus and TotalFill BC Sealer are presented in Table 3. Representative CLSM images of the results for TotalFill BC Sealer are presented in Figure 5.

No significant differences were observed between the tubular penetration depth and percentage of penetration around the canal perimeter between AH Plus sealer and TotalFill BC Sealer (*p* > 0.05), both in total and per third (apical, middle, or coronal).

**Figure 6:**
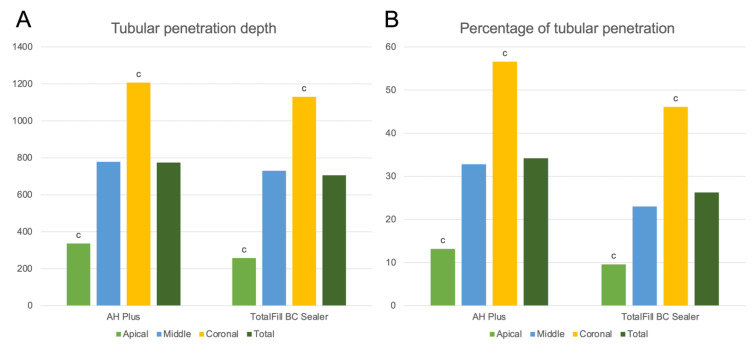
Graphical representation of tubular penetration depth (**A**) and percentage of tubular penetration around the canal perimeter (**B**) for AH Plus and TotalFill BC Sealer. (c) Significant difference between apical and coronal measurement points within each sealer group (*p* < 0.05).

The AH Plus samples showed a significantly different depth of penetration in all measurement points, reducing from the coronal to the apical points (*p* < 0.05). The TotalFill BC Sealer samples, however, only showed a significant reduction in the depth of penetration from the coronal to the apical measurement points (*p* < 0.05) (Figure 6A).

The same was observed with the percentage of tubular penetration around the canal perimeter (*p* < 0.05) (Figure 6B).

### 3.3. Results for TotalFill BC Sealer HiFlow

**Figure 7:**
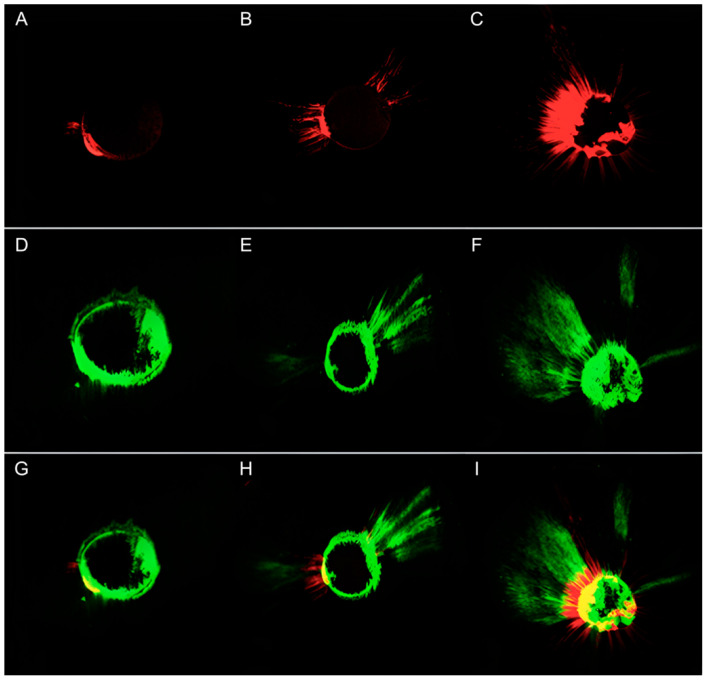
Representative CLSM images of the samples treated with gutta-percha and AH Plus and re-treated with gutta-percha and TotalFill BC Sealer HiFlow. AH Plus: apical point (**A**), medial point (**B**), coronal point (**C**); TotalFill BC Sealer: apical point (**D**), medial point (**E**), coronal point (**F**); both sealers: apical point (**G**), medial point (**H**), coronal point (**I**).

Quantitative results of the CLSM image analysis in terms of tubular penetration depth and percent penetration of the dentinal tubule perimeter exhibited by AH Plus and TotalFill BC Sealer HiFlow are presented in Table 4. Representative CLSM images of the results for the TotalFill BC Sealer HiFlow are presented in Figure 7.

No significant differences were observed between the tubular penetration depth and percentage of penetration around the canal perimeter between AH Plus sealer and TotalFill BC Sealer HiFlow (*p* > 0.05), both in total and per third (apical, middle, or coronal).

**Figure 8:**
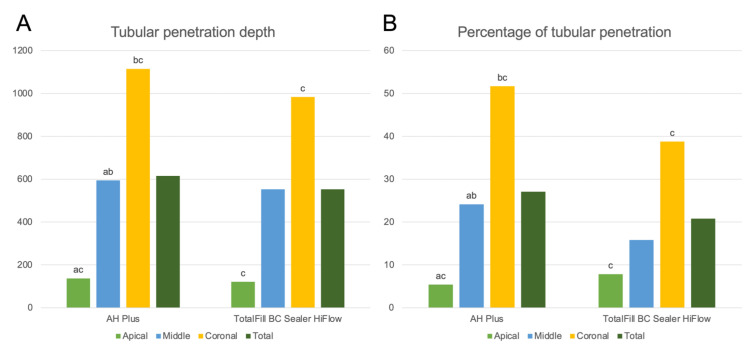
Graphical representation of the tubular penetration depth (**A**) and percentage of tubular penetration around the canal perimeter (**B**) for AH Plus and TotalFill BC Sealer HiFlow. (a) Significant difference between apical and middle measurement points within each sealer group (*p* < 0.05). (b) Significant difference between middle and coronal measurement points within each sealer group (*p* < 0.05). (c) Significant difference between apical and coronal measurement points within each sealer group (*p* < 0.05).

The AH Plus samples showed a significantly different depth of penetration in all measurement points, reducing from the coronal to the apical points (*p* < 0.05). The TotalFill BC Sealer samples, however, only showed a significant reduction in the depth of penetration from the coronal to the apical measurement points (*p* < 0.05) (Figure 8A).

The same was observed with the percentage of tubular penetration around the canal perimeter (*p* < 0.05) (Figure 8B).

## 4. Discussion

The aim of this in vitro study was to assess the intratubular penetration potential of three calcium-silicate-based sealers (CeraSeal, TotalFill BC Sealer, and TotalFill HiFlow) in root canals initially treated with an epoxy-resin-based sealer (AH Plus) via laser confocal optical microscopy. Currently, no endodontic sealer meets all of the expected ideal properties, such as dimensional stability, biocompatibility, and adequate seal [21]. On the other hand, AH Plus has established itself as the reference sealer for comparison with new biomaterials introduced in the market, due to its adequate properties, popularity, and high treatment success rate [5].

The penetration depth of the sealers was evaluated under laser confocal microscopy. It is an observational technique that allows for evaluating the penetration of sealers inside the dentinal tubule using fluorescent staining. This is possible because the microscope performs several slices of the sample at depths of 20–30 µm [22], and overlaps them, providing a three-dimensional image that allows for seeing the path that the sealer has traveled inside the dentinal tubules. In other similar studies, different microscopic techniques have been used to assess tubular penetration, such as optical microscopy [23], scanning electron microscopy [24], or computerized microtomography [25,26]. However, these methods only study the surface of the samples and not their entire extension [15], thus providing limited information compared with confocal laser microscopy.

Regarding the results of the present study, AH Plus sealer showed a higher penetration compared with the three bioceramic sealers; therefore, the null hypothesis was rejected. This was expected, as it was the first sealer used, whereas bioceramic sealers were used for retreatment and thus may have found residual filling materials that hindered their penetration. In addition, one of the reasons for the popular use of AH Plus is its adequate flowability and viscosity, which may result in a higher penetration [14,27]. However, the higher penetration depth and percentage of penetration around the canal perimeter exhibited by AH Plus were not significant (*p* < 0.05) in any of the cases. In other words, the reduction in the penetration of the tested calcium-silicate-based sealers in root canal retreatment is not significant and consequently may not affect the three-dimensional seal of the root canal system negatively.

In parallel, in this study, it was observed that there was a large amount of residual AH Plus sealer inside the dentinal tubules after retreatment with the Reciproc Blue system. This is because, as previously mentioned, no retreatment system completely removes the root canal filling materials that have initially penetrated inside the dentinal tubules [28]. Furthermore, a previously published CLSM study found that AH Plus cement penetrates deeper into dentin than a bioceramic sealer (BC Sealer) [6]. The differences in the tubular penetration of root canal sealers can also be influenced by the differences in their composition. Differences in the format, fillers, and/or thickening agents may account for differences in their physicochemical properties [19]. Nevertheless, as the compositional information regarding the fillers and additives of the tested sealers is not presented in the materials’ respected safety data sheets (SDS) due to confidential business information, these could not be tested in the present study.

Interestingly, the tubular penetration followed the same pattern in all teeth. Both in the depth of penetration in the dentinal tubules and in the percentage of penetration around the canal perimeter, the apical measurement point showed the lowest results, followed by the middle and coronal measurement points. This is in accordance with other authors [12,29]. This may be due to differences in the distribution and diameter of the dentinal tubules according to the position in the root.

This study followed a similar methodology to that of a recently published study by Eğemen et al. [15], in which the effect of root canal treatment on the tubular penetration of calcium-silicate-based sealers during root canal retreatment was assessed. Both in our study and in the aforementioned study, the mean tubular penetration depth and the percentage of penetration around the canal perimeter were evaluated. However, in the study by Eğemen et al., a significant reduction in the penetration depth of the sealers was observed during root canal retreatment. This was also observed in the present study, but the difference was not significant. This difference could be due to the different materials used, the different root canal treatment techniques, or the differences in the anatomical features of the root samples used. Future studies should confirm these results using larger samples and reproducible methodologies.

A limitation of this study lies on the standardization of the quantity of sealer used for the filling of each sample. Nevertheless, the anatomical differences in the root canal systems of the samples intrinsically hinders the standardization process. Another limitation of this study lies in the fact that the same filling method was used for both types of sealers, whereas the manufacturers’ recommendation is that the single cone technique should preferably be applied for calcium silicate sealers. However, this issue is under debate. For example, in the study by DeLong et al. in 2015 [30], the authors concluded that calcium silicate sealers exhibited a lower bond strength when using the continuous wave technique compared with the single cone technique. However, other studies advocate that the continuous wave technique is more effective for filling the lateral canals than the single cone technique [31]. Most recently, a novel hot modified obturation technique has been developed, showing promising results with calcium silicate sealers [32]. In future studies, different techniques should be tested to assess the differences in tubular penetration.

## 5. Conclusions

The results from the present in vitro study report a non-significant reduction in penetration depth and a penetration around the canal perimeter of the calcium-silicate-based sealers CeraSeal, TotalFill BC Sealer, and TotalFill BC Sealer HiFlow in the retreatment of teeth with a prior root canal treatment with the epoxy-resin-based sealer AH Plus. Consequently, this reduction may not affect the three-dimensional seal of the root canal system in a negative manner.

Both the penetration depth and penetration around the canal perimeter were significantly reduced in a coronal−apical manner both in root canal treatment with AH Plus and in the root canal retreatment with the tested calcium-silicate-based sealers.

## Figures and Tables

**Table 1:** Composition of the tested sealers.

Material	Composition
Ceraseal	45–50% zirconium dioxide, 20–30% tricalcium silicate, 1–10% dicalcium silicate, 1–10% tricalcium aluminate
Totalfill BC Sealer	35–45% Zirconium oxide, 20–35% tricalcium silicate, 7–15% dicalcium silicate, 1–4% calcium hydroxide, fillers
Totalfill BC Sealer HiFlow	35–45% Zirconium oxide, 20–35% tricalcium silicate, 7–15% dicalcium silicate, 1–4% calcium hydroxide, fillers
AH Plus	Epoxy paste: diepoxy, calcium tungstate, zirconium oxide, aerosol, and dye Amine paste: 1-adamantane amine, N’dibenzyl-5 oxanonandiamine-1,9, TCD-diamine, calcium tungstate, zirconium oxide, aerosol, and silicone oil

* Data on the composition of the tested sealers were extracted from the materials’ respective safety data sheets (SDS).

**Table 2:** Comparison of the tubular penetration depth and percentage of penetration around the canal perimeter for AH Plus and CeraSeal.

		Tubular Penetration Depth (μm)				Percentage of Penetrated Perimeter (%)			
n = 10		Apical	Middle	Coronal	Total	Apical	Middle	Coronal	Total
AH Plus	Mean	114.04	995.47	1539.83	883.11	4.94	24.11	58.23	29.09
	Median	0.00	769.88	1255.66	706.73	0.00	15.66	63.15	14.73
	IQR	48.79	1386.31	1329.80	1523.15	1.20	32.04	71.08	50.25
CeraSeal	Mean	28.94	663.87	1353.93	682.25	0.67	27.23	49.34	25.75
	Median	0.00	569.08	1228.09	124.42	0.00	15.04	46.48	7.54
	IQR	0.00	1265.33	2237.41	1249.63	0.00	42.58	65.71	42.63

**Table 3:** Comparison of the tubular penetration depth and percentage of penetration around the canal perimeter for AH Plus and TotalFill BC Sealer.

		Tubular Penetration Depth (μm)				Percentage of Penetrated Perimeter (%)			
n = 10		Apical	Middle	Coronal	Total	Apical	Middle	Coronal	Total
AH Plus	Mean	336.74	778.23	1207.08	774.01	13.19	32.81	56.58	34.19
	Median	153.67	839.67	1411.54	777.93	1.73	25.66	66.95	24.18
	IQR	604.97	1162.45	623.06	1383.86	21.58	41.14	55.55	64.50
TotalFill BC Sealer	Mean	257.25	729.51	1130.60	705.79	9.59	23.02	46.11	26.24
	Median	100.55	766.98	1218.06	683.17	9.81	19.71	49.20	16.41
	IQR	490.11	1134.02	915.81	1203.22	15.94	27.81	66.23	34.90

**Table 4:** Comparison of the tubular penetration depth and percentage of penetration around the canal perimeter for AH Plus and TotalFill BC Sealer.

		Tubular Penetration Depth (μm)				Percentage of Penetrated Perimeter (%)			
n = 10		Apical	Middle	Coronal	Total	Apical	Middle	Coronal	Total
AH Plus	Mean	137.26	594.11	1114.95	615.44	5.39	24.15	51.69	27.07
	Median	0.00	622.22	1308.77	605.52	0.00	26.63	52.89	24.95
	IQR	283.69	679.61	742.33	1181.20	10.92	32.69	36.14	50.55
TotalFill BC Sealer HiFlow	Mean	121.35	552.51	983.84	552.57	7.85	15.81	38.77	20.80
	Median	0.00	293.62	1029.81	293.62	0.00	9.11	34.83	9.11
	IQR	104.68	1350.94	882.79	1202.03	9.50	29.33	54.77	40.86

## Data Availability

The data presented in this study are available upon request from the corresponding author.
